# Dependence of Bacterial OTUs on Selected Features of Beaver Ponds

**DOI:** 10.1002/ece3.72790

**Published:** 2025-12-25

**Authors:** Michał Wróbel, Radosław Gawryś, Anna Tereba, Magdalena Frąk, Katarzyna Sikora, Karol Sokołowski, Andrzej Boczoń

**Affiliations:** ^1^ Department of Forest Ecology, Forest Research Institute Sękocin Stary Poland; ^2^ Department of Remote Sensing and Environmental Assessment, Warsaw University of Life Sciences Warsaw Poland

**Keywords:** DCA analysis, European beaver ( *Castor fiber* L.), metagenomic analysis, OTUs

## Abstract

Ponds created by beavers represent unique aquatic ecosystems that influence hydrological, chemical and biological conditions, including the microbiology of the water. The activity of these animals promotes biodiversity and water purification processes, but can also lead to the accumulation of pollutants. Water retention in beaver ponds promotes the development of bacteria and other microorganisms that play an important role in biogeochemical cycles. Long‐term water stagnation can lead to anaerobic conditions and the formation of toxic compounds, which in turn can limit the diversity of benthic organisms. Beavers play a key role in shaping these habitats, and microbiological studies of their reservoirs provide a better understanding of their impact on aquatic ecosystems, self‐purification processes and potential biological threats. Metagenomic analysis revealed the presence of 365 bacterial species in water and sediment samples, belonging to 174 genera and 83 families. 83 operational taxonomic units (OTUs) were identified, 62 of which were present in both water and sediments. Although the overall OTU composition was similar in both environments, greater variability was observed in the sediments. The statistical differences in OTU distribution between water and sediments were confirmed using the Wilcoxon test.

## Graphical abstract

Beaver ponds create unique aquatic ecosystems that enhance biodiversity and support water purification but can also contribute to pollutant accumulation and anaerobic conditions. A total of 83 operational taxonomic units (OTUs) were identified, of which 62 were present in both water and sediment samples, indicating a high degree of microbiological similarity between these environments. These findings highlight the ecological importance of beavers in shaping microbial diversity and ecosystem processes in freshwater environments.

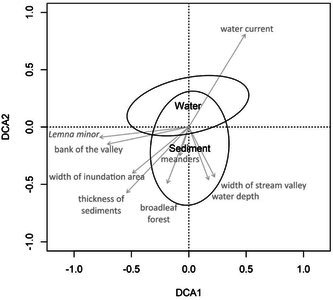

## 1 Introduction

Ponds created by beavers are unique aquatic ecosystems created by the construction of dams and flooding. These changes affect hydrological, chemical and biological conditions while altering the microbiological composition of the water, including the presence of different bacterial species (Rolauffs et al. 2001; Bush and Wissinger 2016; Vorel 2016; Baker and Hill 2003; Czech 2000). The water retention caused by beaver activity helps to increase biodiversity and supports water purification processes, but can also lead to the accumulation of organic matter, which can result in the accumulation of potentially harmful pollutants (Schwab 2009; Campbell‐Palmer and Rosell 2013). The effects of these changes on microbiological processes in water and bottom sediments are complex and require further research (Rozhkova‐Timina et al. 2018). Water ecosystems in the forest environment may contain both substances of anthropogenic origin and compounds resulting from natural processes in the catchment. A large amount of plant material introduced into the ecosystem, especially with limited water circulation, can lead to increased saprobic activity and the creation of anaerobic conditions. As a result, the process of decomposition of organic matter dominates and compounds with toxic properties for aquatic organisms, especially those living in the bottom zone, may also occur (Collen and Gibson 2001; McDowell and Naiman 1986). Structures built by beavers significantly affect the activity of microorganisms along streams and lead to changes in the biogeochemical cycles regulated by microorganisms (Songster‐Alpin and Klotz 1995). The slowing of the water flow promotes the sedimentation of suspensions and organic matter, which creates favorable conditions for the growth of bacteria. Microorganisms involved in biodegradation processes play a key role in biogeochemical cycles, and their diversity is an important element in the study of microbial community dynamics in aquatic ecosystems (Behera et al. 2021). Microorganisms occur in large numbers in the reservoirs created by beavers due to the presence of abundant organic material from vegetation and animal remains. Over time, such reservoirs can turn into wetlands that act as natural sewage treatment plants. The microorganisms living there are involved in the decomposition of organic and inorganic substances and thus contribute to improving water quality. Studies on the bacteria present in beaver ponds allow a better understanding of their impact on aquatic ecosystems, especially in relation to self‐purification processes and changes in microbiological composition. Monitoring the dynamics of bacterial populations enables the assessment of water quality and the identification of potential threats from pathogens or biological pollutants. Beavers play a key role in shaping the aquatic environment, while microorganisms play an important role in regulating biogeochemical processes and maintaining the ecological balance of these ecosystems. As only a small percentage of bacteria can be identified using conventional culture methods, the metagenomic approach is increasingly being used to study the microbiology of beaver ponds. This modern technique enables a comprehensive analysis of microbial communities in aquatic ecosystems and provides valuable information on their dynamics and effects on water quality (Amann et al. 1995; Handelsman et al. 1998; Behera et al. 2021; Cabello‐Yeves et al. 2021; Nguyen et al. 2022; Ngugi et al. 2023).

Traditional microbiological methods, based on culture and microscopic identification, have significant limitations in studying aquatic environments. Only a small percentage of microorganisms present in environmental samples can be cultured in the laboratory, as most aquatic bacteria require specific physicochemical conditions that are difficult to reproduce outside their natural environment (Amann et al. 1995; Hugerth and Andersson 2017). Similarly, classical biochemical techniques or PCR targeting selected taxonomic groups allow analysis of only a fraction of the microbial community. These limitations hinder a comprehensive understanding of the diversity and ecological functions of microorganisms in complex ecosystems such as beaver ponds. Metagenomics overcomes these limitations by enabling direct DNA sequencing, allowing detection of both dominant and rare taxa, understanding community structure and dynamics, and identifying potentially novel, unculturable microorganisms. Metagenomics is therefore a modern and effective tool for studying microbial diversity in various ecosystems and determining the relationships between environmental characteristics and the structure of bacterial communities (Amann et al. 1995; Handelsman et al. 1998; Behera et al. 2021; Cabello‐Yeves et al. 2021; Nguyen et al. 2022; Ngugi et al. 2023).

The main aim of this study was to determine how selected environmental and geomorphological characteristics of beaver ponds influence the composition and diversity of bacterial taxonomic units (OTUs) in water and sediments. The microbial component of ecosystems created by beavers is still relatively poorly understood, despite the key role of microorganisms in element cycling, organic matter decomposition, and water self‐purification. Beaver ponds are dynamic, semi‐natural water bodies where microbiomes are shaped by unique hydrological and physicochemical gradients created by beaver activity. Studying microbial diversity in these environments is therefore essential to understanding how beaver ecosystem engineering influences the functioning of aquatic ecosystems and contributes to increased biodiversity at the landscape scale. The aim of this study was to characterize the diversity and composition of bacterial communities in beaver ponds using 16S rRNA sequencing, compare the taxonomic structure and diversity of bacterial communities between water and sediment samples, assess the influence of key environmental and geomorphological factors on microbial community structure, and identify potential relationships between environmental parameters and dominant bacterial taxa that may explain the ecological functioning of beaver ponds.

## 2 Methods

**FIGURE 1:**
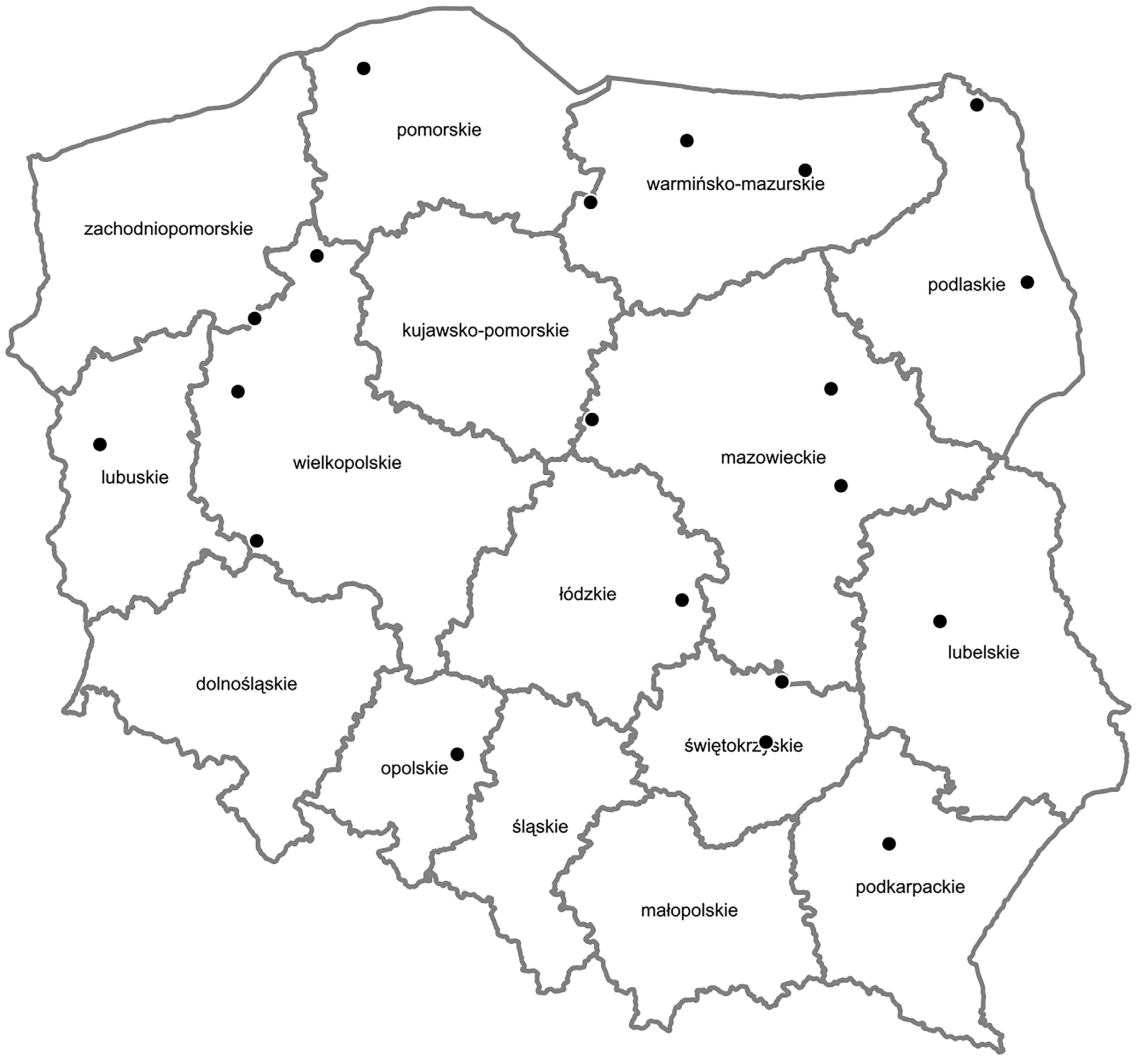
Map of the distribution of research areas in the context of the voivodeships.

The study was carried out at 20 sites in forest areas in various regions of Poland (Figure 1). The investigated objects are water reservoirs created by the activity of beavers, which have dammed up water in streams by building dams. When selecting the study sites, the age of the beaver structures was taken into account—only those that had been in existence for at least 7 years were selected. Such a long period of operation of the structures ensured a stable accumulation of water, which favored long‐term deposition of organic material and maintained the appropriate depth of the reservoir, sediment thickness and width of the valley, as well as inundation area. Samples for analysis were collected during the surface period from 30 May to 9 June. To determine the chemical composition of the water, samples were taken from depths corresponding to 10% and 50% of the total pool depth at three locations in the beaver pond. One composite sample was prepared and transported to the laboratory in a sterile bottle at +4°C. Sediment samples were collected by core sampling from three locations in the beaver pond, and the composite sample was transported to the laboratory under the same conditions as the water samples. As part of the analyzes carried out between the juxtaposition of possible relationships between the chemistry of the water and the sediments, selected characteristics of the studied objects were determined using the expert method. These are water depth, sediment thickness, valley width, width of inundation area. In addition, the objects (beaver ponds) were categorized according to the flow, meandering, occurrence of *Lemna minor* , forest habitat and slope of the valley banks using the expert method. For each category, a group was determined that defines the respective category (Table 1).

**TABLE 1:** Categories of objects with the group designation.

Category	Group	Description
Water current	0	None
	1	Occurrence
Meanders	0	Regulated stream
	1	Natural stream
*Lemna minor*	0	None
	1	Occurrence
Broadleaf forest	0	None
	1	Occurrence
Bank of the valley	0	Valley bank slope < 30^o^
	1	Valley bank slope > 30^o^

The investigation of bacterial populations using the metagenomic method was carried out by analyzing the hypervariable V3‐V4 region of the 16S rRNA gene. 16S rRNA sequencing is often used as an important tool for the taxonomic classification of microorganisms (Woese 1987; Johnson et al. 2019). The specific primers 341F and 785R (16S analysis) were used to amplify the selected fragment and create a library. PCR was performed using the Q5 Hot Start High‐Fidelity 2× Master Mix according to the manufacturer's guidelines. Sequencing was performed on the MiSeq platform with paired‐end (PE) technology, 2 × 300 nt, using Illumina's v3 kit. Initial data analysis was performed automatically on the MiSeq instrument using MiSeq Reporter (MSR) v2.6 software. Bioinformatic data analysis, allowing classification of reads to species level, was performed using the QIIME 2 package based on the Silva 138 reference sequence database. In addition, the DADA2 package was used to separate sequences of true biological origin from those generated during sequencing. This package was also used to identify unique amplicon sequence variants (ASVs). The statistical analysis included the identified bacterial families and used the operational taxonomic unit (OTU), which groups organisms with similar DNA sequences of the marker gene (Morata de Ambrosini et al. 1999; Vane‐Wright 2001; Blaxter et al. 2005; Bhargava and Sharma 2013). OTUs are practical equivalents of species at different taxonomic levels in the case of microorganisms for which traditional classification systems are less precise. When analyzing 16S rRNA (prokaryotes) or 18S rRNA (eukaryotes) marker genes, sequences are grouped based on their mutual similarity, with a similarity threshold set by the researcher. There is an ongoing debate about the extent to which this method reflects the true phylogenetic and ecological relationships between microorganisms. Although the method of OTU delineation can vary depending on the algorithm and threshold, the study by Schmidt et al. (2014) showed that the OTU structure is generally stable across different environments and clustering methods. The statistical analysis was performed in R. Bacterial diversity at the family level between the substrates studied was visualized using DCA analysis (function “decorana”, package “vegan”). In addition, the relationship between the arrangement of the beaver ponds and their characteristics was determined using the “envfit” function from the “vegan” package. *p*‐values < 0.05 were considered statistically significant.

## 3 Results

The assumed properties of the objects were described and measured. The average water depth in the investigated beaver ponds was 61 cm and ranged from 20 cm to 120 cm. The greatest differences in water depth occurred in the category of the “meander” pond division, which combines ponds on streams with regulated and simplified channels and ponds on unregulated or renaturalized streams with meanders. The average organic sediment thickness was 30.3 cm and ranged from 0 cm to 80 cm. The greatest differences in sediment thickness occurred between groups differentiated by the presence of a current—ponds with a stream had almost half the sediment thickness. The width of the valley in which the beaver ponds were located averaged 33.5 m, with this value ranging from 5 m to 60 m for individual sites. The greatest differences in the average value of this feature occurred between groups distinguished by the angle of the pond banks—the width of valleys with gently sloping pond banks was almost twice as high as for ponds with banks bounded by steep slopes.

The width of the inundation caused by beavers through the construction of dams was 14.2 m on average. The greatest differences in the average value of this feature occurred between groups differentiated according to the presence of the current—ponds with its presence were almost half as wide.

The values of individual characteristics as a function of category and number of sites are shown in Table 2.

**TABLE 2:** Descriptive statistics of selected features of beaver ponds.

Variable	Category	Group	*N*	M	SE	Min	Me	Max
Water depth (cm)	Total		20	61.0	6.2	20	60	120
	Water current	0	9	58.9	11.5	20	50	120
	Water current	1	11	62.7	6.9	30	65	100
	Meanders	0	12	67.1	9.6	20	65	120
	Meanders	1	8	51.9	5.0	30	55	70
	*Lemna minor*	0	9	55.0	7.2	20	60	90
	*Lemna minor*	1	11	65.9	9.7	30	60	120
	Broadleaf forest	0	10	62.0	8.8	30	55	120
	Broadleaf forest	1	10	60.0	9.3	20	60	110
	Bank of the valley	0	10	60.5	9.0	30	55	120
	Bank of the valley	1	10	61.5	9.1	20	60	110
Organic sediments thickness (cm)	Total		20	30.3	4.6	0	30	80
	Water current	0	9	40.0	8.7	0	40	80
	Water current	1	11	22.3	3.0	10	20	40
	Meanders	0	12	32.5	6.6	0	30	80
	Meanders	1	8	26.9	5.9	0	27.5	50
	*Lemna minor*	0	9	22.2	6.2	0	20	60
	*Lemna minor*	1	11	36.8	6.1	0	40	80
	Broadleaf forest	0	10	33.5	7.1	0	35	80
	Broadleaf forest	1	10	27.0	6.0	0	25	60
	Bank of the valley	0	10	32.0	7.9	0	35	80
	Bank of the valley	1	10	28.5	5.1	0	27.5	60
Width of valley (m)	Total		20	33.5	4.2	5	30	80
	Water current	0	9	38.9	7.5	10	30	80
	Water current	1	11	29.1	4.4	5	25	50
	Meanders	0	12	32.9	6.2	5	27.5	80
	Meanders	1	8	34.4	5.3	15	35	50
	*Lemna minor*	0	9	26.1	4.5	5	25	50
	*Lemna minor*	1	11	39.5	6.3	10	40	80
	Broadleaf forest	0	10	37.0	5.9	20	30	80
	Broadleaf forest	1	10	30.0	6.0	5	25	60
	Bank of the valley	0	10	42.0	6.8	5	45	80
	Bank of the valley	1	10	25.0	3.5	10	22.5	50
With of inundation (m)	Total		20	14.2	2.5	2	11	40
	Water current	0	9	20.0	4.4	3	20	40
	Water current	1	11	9.4	2.1	2	5	20
	Meanders	0	12	11.9	3.5	2	5	40
	Meanders	1	8	17.5	3.3	5	17.5	30
	*Lemna minor*	0	9	13.1	3.1	3	15	30
	*Lemna minor*	1	11	15.0	3.9	2	10	40
	Broadleaf forest	0	10	14.5	3.2	3	13.5	30
	Broadleaf forest	1	10	13.8	4.0	2	9	40
	Bank of the valley	0	10	14.4	4.4	3	6.5	40
	Bank of the valley	1	10	13.9	2.8	2	13.5	30

Abbreviations: M, arithmetic mean; Max, maximum; Me, median; Min, minimum; *N*, number of cases; SE, standard error.

The metagenomic analysis revealed a total of 365 bacterial species in water and sediment samples, distributed across 174 genera, 83 families, 36 orders, 12 classes and 8 phyla. The genetic analyzes carried out focused on bacterial families, as these could be identified quite well. 83 bacterial families were identified in the collected samples. 83 OTUs (operational taxonomic units) of bacteria (families) were found in the collected samples, 79 in sediment samples and 66 in water samples. 62 OTUs were found in both substrates. Both the average number of OTUs and the average value of the Shannon‐Wiener index in the sediment and water samples were similar (Table 3) and showed no statistically significant differences, as shown by the Wilcoxon test at *p* = 0.05.

**TABLE 3:** Descriptive statistics of the Shannon‐Wiener diversity index and the number of OTUs (families) in water and sediment samples.

Variable	Group	*n*	Mean	SD	Min	Q1	Median	Q3	Max
Shannon‐Wiener Index	Sediment	20	1.95	0.28	1.31	1.84	1.99	2.11	2.39
	Water	20	1.95	0.45	1.05	1.69	2.06	2.20	2.72
Number of OTUs	Sediment	20	27.80	5.42	16.00	25.00	27.00	31.50	37.00
	Water	20	27.30	7.43	16.00	20.75	27.50	34.00	41.00

**FIGURE 2:**
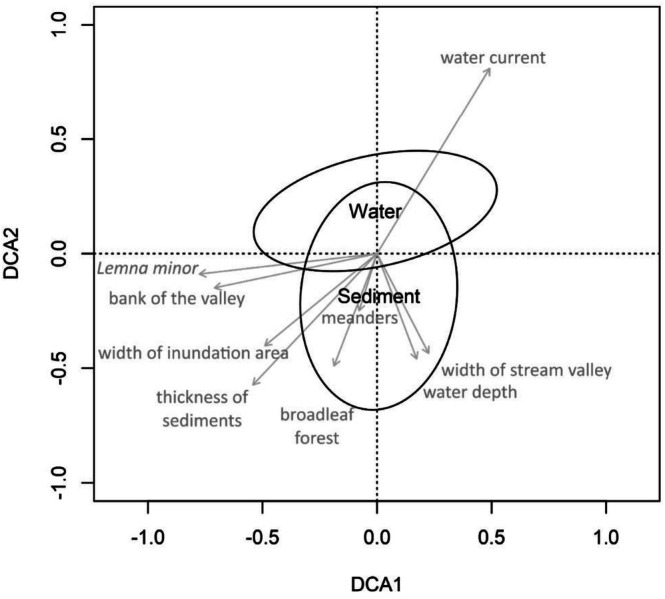
The result of the DCA analysis shows the relationship between the composition of OTUs (families) in water and sediment samples (ellipse = 1SD) and selected features of the beaver ponds. DCA1 = 0.285; DCA2 = 0.215. H_water—water depth, H_sediment—sediment thickness. D_flood—flood width, D_valley—valley width.

The composition of OTUs in water and sediment samples was based on a similar set of taxa. However, the location in the ordination space of water and sediment samples from the same ponds differed significantly on both the DCA1 (*p* < 0.001) and DCA2 (*p* = 0.021) axes according to the Wilcoxon test. In addition, the composition of bacterial OTUs from the sediments showed greater variability than the composition of bacterial OTUs in the water (Figure 2). In the DCA ordination space, sediment samples showed a broader distribution than water samples, indicating greater variation in bacterial community composition in sediment sites. In contrast, water samples formed a more compact cluster, suggesting a relatively homogeneous microbial structure in the water column. The separation of the two groups along the first ordination axis reflects distinct differences in the composition of bacterial communities associated with sediments and water. Additionally, samples from flowing reservoirs tended to cluster separately from those from stagnant reservoirs, indicating that hydrological conditions influence the structure of microbial communities.

## 4 Summary

Bacteria play a key role in aquatic ecosystems. They are reducers, so to speak, involved in the decomposition and cycling of organic matter (Newton et al. 2011). In this article, indicator bacteria families for water and sediments of beaver ponds are presented. Beaver ponds are unique ecosystems in which beaver activities such as dam construction transform streams into floodplains, affecting hydrological, chemical and biological conditions. These changes affect the microbiological composition of waters, especially bacteria. Although Water retention increases biodiversity and supports water purification, it can also lead to the accumulation of pollutants. The microorganisms in such ponds play a key role in biogeochemical processes, but long‐term stagnation can favor the development of toxic conditions, which limits the diversity of fauna.

The results of the analyses showed that the physical characteristics of beaver ponds exhibit considerable variability, both in terms of hydromorphological conditions and the presence of habitat features such as the presence of *Lemna minor* or the presence of forest. These variables can significantly influence the environmental conditions that determine the composition of the water and sediment microbiome. In addition, environmental analysis has shown that the greatest differences in characteristics such as water depth, sediment thickness, valley or flood width occur depending on the presence of the stream and the degree of stream meandering, confirming the influence of local hydrological conditions on pond structure (Naiman et al. 1986; Rosell et al. 2005). The water depth in the studied ponds was highly variable (20–120 cm), with an average of 61 cm. The shallowest ponds were observed in areas with meanders and with the presence of *Lemna minor* , which could indicate greater plant succession and stable water conditions. Water depth is an important factor influencing oxygen supply, light access and temperature. These factors in turn determine the development of microbial communities and biogeochemical processes (Johnston and Naiman 1990). The thickness of the bottom sediments also varied (0–80 cm) and reached the highest values in ponds without current (average 40 cm) and in the presence of *Lemna minor* (average 36.8 cm). A thicker sediment layer under such conditions may indicate a slower flow and an accumulation of organic material, which favors the development of anaerobic microbiological processes. In turn, a thinner sediment layer in ponds with a current may be due to mechanical flushing and lower retention. The width of the valley and the width of the inlet reflect morphological features of the landscape that influence water retention and the development of the littoral zone. Wider valleys and inlets were observed in ponds without bank vegetation, which may indicate greater anthropogenic influence or natural spatial openness. Reducing the width of valleys and inlets in dense vegetation and near forest may affect shading, microclimate, and habitat stability (Hood and Larson 2015). The variability of these physical parameters not only shapes the hydrological and chemical conditions of beaver ponds, but also underlies differences in the composition and structure of the sediment and water microbiome. In addition, the influence of beavers as ecosystem engineers, which manifests itself in the creation of meanders, the damming of water or the increase in the thickness of sediments, among other things, leads to the creation of a mosaic landscape of habitats that increases spatial heterogeneity and biodiversity (Naiman et al. 1986; Stringer and Gaywood 2016).

The metagenomic analysis carried out in this study revealed a considerable bacterial diversity in the water and sediment samples analyzed, comprising a total of 365 species belonging to 174 genera, 83 families, 36 orders, 12 classes and 8 phyla. Focusing on the family level (83 OTUs) proved to be a good choice, as this taxonomic level represents a reasonable compromise between resolution and identification confidence in metagenomic analyses (Hugerth and Andersson 2017). Using 16S rRNA sequencing and metagenomic analysis, we showed that the main environmental factors shaping microbial communities in beaver ponds were the presence or absence of flow, bottom sediment thickness, valley morphology, and vegetation cover. Ponds with weak or absent flow and greater sediment thickness exhibited greater variability and taxonomic richness of bacteria in the sediments, indicating the development of anoxic microzones and intensive organic matter decomposition. Water samples showed less variability, suggesting a more homogeneous and oxygenated microbial environment. The results showed that the structure of bacterial diversity at the family level was comparable between water and sediment samples, both in terms of the average number of OTUs and the Shannon‐Wiener index. No statistically significant differences (*p* > 0.05) were found between bacterial communities in water and sediment; however, this does not indicate that both environments have the same microbial composition. Despite the similar OTU composition, the distribution of samples in the ordination chamber (DCA) shows significant differences in microbial community structure between water and sediment (DCA1: *p* < 0.001; DCA2: *p* = 0.021). This suggests that while the overall taxonomic composition may be similar, differences in the dominance of individual families or their relative abundance lead to a clear ecological separation. This is consistent with observations suggesting that sediment and water microbiomes differ functionally and structurally, often in response to local physicochemical conditions and organic matter availability (Zhou et al. 2017). These findings highlight the importance of analyzing the microbiome in both water and sediment samples to obtain a more complete picture of the ecosystem. Despite the similarities in taxonomic richness, differences in structure may have important implications for biogeochemical processes and aquatic ecosystem health. The sediment samples showed greater variability in composition, which may be due to the greater heterogeneity of the benthic environment. Bottom sediments of water reservoirs, especially those formed by beaver activities, are characterized by complex physicochemical structure, variable organic matter availability and variable oxygen ratios (Ye Deng et al. 2012; Battin et al. 2016). This suggests that sediments represent a more stable but internally diverse environment that can support greater microbiological diversity. Common OTUs (62 OTUs) were found in both sediments and water, confirming the existence of a common microbial core for both types of environments, but differences in their relative proportion indicate the selective influence of local environmental conditions. Similar observations were made by Zwart et al. (2002), among others, who pointed to differences in the benthic microbiome in freshwater lakes. The presence of meanders and forests can promote the stabilization of hydrological conditions and increase the retention of organic matter, which in turn influences the development of certain bacterial communities (Naiman et al. 1986). Greater water depth and weaker flow, in turn, can limit the mixing of water layers, which favors the development of anaerobic and fermentative microorganisms (Shade et al. 2012). From an ecological perspective, the results indicate that beaver ponds are environments with high microbiological heterogeneity, resulting from the mosaic structure of their habitats—from aerobic conditions in the water column to anaerobic conditions in the bottom sediments. This structure enables the coexistence of diverse functional groups of bacteria involved in key biogeochemical processes, including the nitrogen, phosphorus, and carbon cycles. The observed diversity of microbial communities is ecologically significant—diverse bacterial communities contribute to nutrient retention, pollutant removal, and increased water self‐purification capacity. Therefore, beavers function as ecosystem engineers not only on a macroscale but also as indirect regulators of biogeochemical processes, enhancing the resilience and functional complexity of forest aquatic ecosystems. Compared to other small water bodies, such as anthropogenic or mid‐field ponds, beaver ponds exhibit greater variability in hydrological conditions, higher organic matter accumulation in sediments, and a significant presence of anaerobic and saprophytic bacteria, reflecting the natural character of these ecosystems and the absence of direct anthropogenic pressure. These factors lead to increased bacterial diversity and functional complexity. Previous studies (Behera et al. 2021; Zhou et al. 2017; Zhang et al. 2021) have shown that microbial diversity in stagnant or semi‐stagnant beaver ponds can exceed that observed in typical forest or agricultural ponds.

The results of this study confirm this trend—despite similar total OTU richness in water and sediments, metagenomic analysis revealed a distinct community structure, reflecting ecological specialization and adaptation of microorganisms to the varying microenvironments in beaver ponds. The applied approach combining environmental and metagenomic analyses provides a comprehensive understanding of the role of beavers in shaping biodiversity, both macro‐ and microbiological, and can provide a basis for further research on the effects of biological engineering on the functioning of small aquatic ecosystems. In the context of ecosystem functioning, the presence of beavers has a significant impact on the microbiological diversity of ponds by altering the hydrological and geomorphological structure of the environment. As studies of river and pond ecosystems altered by beavers have shown (Rosell et al. 2005), beaver activities can create ecological niches that support diverse bacterial communities. Understanding how environmental factors and hydromorphological changes caused by beaver activity affect bacterial communities will enable a better assessment of the ecological functioning and resilience of these systems. This knowledge has fundamental implications for microbial ecology and practical implications for water resource management, biodiversity conservation, and the restoration of small reservoirs.

While this study may provide new information on microbial diversity and the ecological significance of beaver ponds, certain limitations must be acknowledged. The analysis relied primarily on 16S rRNA sequencing, which allows for taxonomic identification but offers limited insight into microbial functions. Therefore, the assigned ecological functions are indirect. Future studies should use more advanced methods to more precisely identify the microbial interactions responsible for biogeochemical processes in beaver ponds. Furthermore, the study was limited in both spatial and temporal scope, covering a small number of ponds and a single sampling season. Microbial communities in beaver ponds can exhibit significant seasonal and hydrological variability. Therefore, long‐term monitoring across different climatic conditions and catchments is crucial to capture temporal dynamics and more comprehensively assess the impact of beaver activity on microbiome structure. The physicochemical and geomorphological parameters analyzed in this study reflect only part of the heterogeneity of beaver pond environments. Including additional variables in future studies, such as sediment chemical composition, organic matter characteristics, and microbial functional traits, would provide a better understanding of the complex relationships between habitat structure and ecosystem functioning. In summary, future studies combining advanced molecular approaches with thorough hydromorphological and biogeochemical analysis will be essential to fully understand the role of beavers as ecosystem engineers, shaping both microbial diversity and key ecosystem processes.

## Author Contributions

**Michał Wróbel:** conceptualization (equal), data curation (equal), formal analysis (equal), funding acquisition (lead), methodology (equal), project administration (lead), writing – original draft (lead), writing – review and editing (lead). **Radosław Gawryś:** conceptualization (equal), data curation (equal), formal analysis (equal), methodology (equal), software (equal), validation (lead), writing – review and editing (supporting). **Anna Tereba:** data curation (equal), methodology (supporting), validation (supporting), writing – review and editing (equal). **Magdalena Frąk:** conceptualization (equal), data curation (equal), methodology (equal), resources (equal). **Katarzyna Sikora:** conceptualization (supporting), data curation (supporting), methodology (equal), resources (supporting). **Karol Sokołowski:** investigation (equal), software (supporting), validation (supporting), writing – review and editing (supporting). **Andrzej Boczoń:** formal analysis (supporting), methodology (supporting), validation (supporting), writing – review and editing (supporting).

## Funding

This work was financed from the Forest Research Institute's Research Fund (project no. 260126).

## Conflicts of Interest

The authors declare no conflicts of interest.

## Data Availability

The sequences have been deposited in the NCBI database under numbers: SAMN50085966–SAMN50086005 and BioProject accession number—PRJNA1294181.
